# Accuracy of Time to Treatment Initiation Data in Musculoskeletal Soft Tissue Sarcoma

**DOI:** 10.1155/2023/9022770

**Published:** 2023-05-23

**Authors:** Joshua M. Lawrenz, Jose F. Vega, Jaiben George, Gannon L. Curtis, Jaymeson Gordon, Amanda Maggiotto, Katherine Tullio, Dale R. Shepard, John D. Reith, Herbert S. Schwartz, Lukas M. Nystrom, Nathan W. Mesko

**Affiliations:** ^1^Department of Orthopaedic Surgery, Vanderbilt University Medical Center, Nashville, TN 37232, USA; ^2^Department of Orthopaedic Surgery, Cleveland Clinic, Cleveland, OH 44195, USA; ^3^Department of Orthopaedic Surgery, AIIMS Hospital, New Delhi, India; ^4^Department of Urology, University Health Center, Wayne State University, Detroit, MI 48201, USA; ^5^Case Western Reserve University School of Medicine, Cleveland, OH 44106, USA; ^6^Taussig Cancer Center, Cleveland Clinic, Cleveland, OH 44195, USA; ^7^Department of Pathology, Cleveland Clinic, Cleveland, OH 44195, USA

## Abstract

**Background:**

Time to treatment initiation (TTI) is a quality metric in cancer care. The purpose of this study is to determine the accuracy of TTI data from a single cancer center registry that reports to the National Cancer Database (NCDB) for sarcoma diagnoses.

**Methods:**

A retrospective analysis of a single Commission on Cancer (CoC)-accredited cancer center's tumor registry between 2006 and 2016 identified 402 patients who underwent treatment of a musculoskeletal soft tissue sarcoma and had TTI data available. Registry-reported TTI was extracted from the tumor registry. Effective TTI was manually calculated by medical record review as the number of days from the date of tissue diagnosis to initiation of first effective treatment. Effective treatment was defined as oncologic surgical excision or initiation of radiation therapy or chemotherapy. Registry-reported TTI and effective TTI values were compared for concordance in all patients.

**Results:**

In the entire cohort, 25% (99/402) of patients had TTI data discordance, all related to surgical treatment definition. For patients with a registry-reported value of TTI = 0 days, 74% (87/118) had a diagnostic surgical procedure coded as their first treatment event, with 73 unplanned incomplete excision procedures and 14 incisional biopsies. In these patients, effective TTI was on average 59 days (*P* < 0.001). For patients with a registry-reported value of TTI >0 days, only 4% (12/284) had discordant TTI values.

**Conclusions:**

Nearly three-fourths of patients with a registry-reported value of TTI = 0 days in a large, CoC-accredited cancer center registry had a diagnostic procedure coded as their first treatment event, though their effective treatment had not yet started. These data suggest that TTI is likely longer than what is reported to the NCDB. Redefinition of what constitutes surgical treatment should be considered to improve the accuracy of data used in measuring TTI in sarcoma.

## 1. Introduction

Time to treatment initiation (TTI), defined as the time from diagnosis to initiation of first treatment, is a quality metric some cancer centers have adopted with intention to improve patient experience and survival. Though not a Commission on Cancer (CoC) quality measure, TTI is a datapoint that is reported using the National Cancer Database (NCDB). The association of TTI with patient demographic factors and survival has been reported for several cancer types, including breast and head and neck cancer [1, 2]. More recently, the current national median, trends in TTI, and its association with patient factors and survival in soft tissue sarcoma have been reported [3, 4]. From 2004 to 2013, the national median TTI was 22 days, with a transition in care between institutions being correlated with the greatest delay in TTI [3]. Delays in TTI greater than six weeks showed a trend toward decreased survival [4].

A unique aspect of a tertiary soft tissue sarcoma practice is the large percentage (24–60%) of patients that have undergone unplanned incomplete excisions, also known as “whoops” procedures, prior to referral [5–9]. The “unplanned” nature of the excision is not in reference to the occurrence of the surgery itself, but rather to the lack of recognition on the part of the surgeon prior to the surgery that he or she may be operating on a sarcoma. The deleterious effects of a “whoops” procedure are well described, often resulting in additional surgeries, increased cost, and changes in treatment course [9–11]. According to the current NCDB definition of the “time period for the first course of treatment” or TTI, the date of any “first surgical procedure” qualifies in that calculation [12]. Thus, when a “whoops” procedure occurs, the tumor is both diagnosed (histologic evaluation) and first treated (excision) on the same day based on that definition. Regardless of the oncologic effectiveness of the surgery, current national coding standards would classify this case as TTI = 0 days. With the prevailing assumption that shorter TTI is good, this would represent the best possible scenario if using TTI as a quality metric. On the contrary, we argue that an unplanned incomplete excision procedure or a “whoops” procedure is a diagnostic procedure, not a therapeutic procedure, and the first treatment date that should be used in calculating that patient's TTI value is the date of the patient's first effective treatment.

Familiarity with our cancer center registry data and noticing trends, such as the scenario described, have led to the current study. The purpose of this study is to assess the accuracy of TTI data in soft tissue sarcoma patients in a CoC-accredited cancer center's tumor registry. In doing so we aim to (1) measure and compare the registry-reported and effective TTI values and (2) report the reasons for discordant TTI values.

## 2. Methods

### 2.1. Study Design and Patient Selection

**Figure 1:**
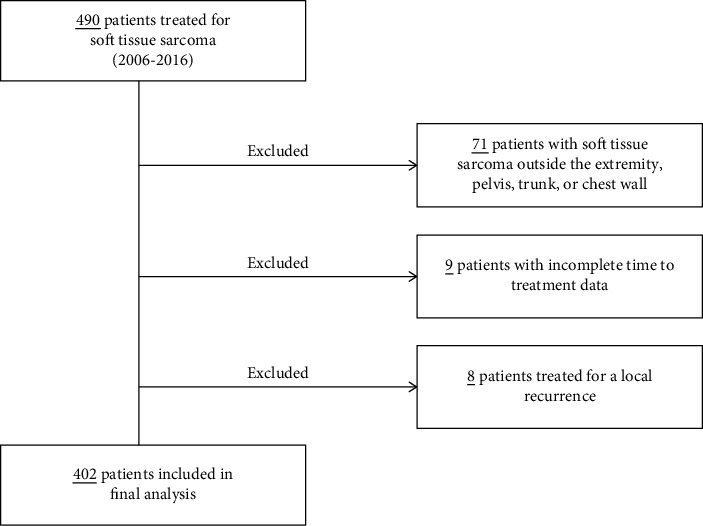
Patient inclusion and exclusion criteria.

Following approval from our institutional review board, a retrospective analysis of our CoC-accredited tumor registry was performed for all soft tissue sarcoma patients between January 2006 and December 2016. A total of 490 patients who received their index treatment at our institution for a soft tissue sarcoma were initially identified, and 71 were excluded for tumors outside the extremity, pelvis, trunk, or chest wall. Of the remaining 419 patients, 17 were excluded for either incomplete TTI data (9 patients) or for the entry being related to treatment of a known local recurrence (8 patients). Thus, 402 patients with a primary soft tissue sarcoma of the extremity, pelvis, trunk, or chest wall and complete time to treatment data were eligible for inclusion in this analysis. Patients that received consultation followed by neoadjuvant radiation or chemotherapy treatment at a different institution and then returned for their surgical excision event at our institution were not included. The inclusion and exclusion criteria of the study cohort can be found in Figure 1.

### 2.2. Data Collection, Definitions, and Outcome

The tumor registry provided patient demographics (age, sex, race, insurance status, and state of residence), tumor characteristics (histology, location, grade, and stage), and treatment information (date of diagnosis, date of first treatment, type of first treatment, and type of physician who performed first treatment). The type of treatment included surgical excision, radiation, or chemotherapy. The type of physician who performed treatment was specified in our registry as a sarcoma-specialized physician within our health system, nonsarcoma physician within our health system, or outside health system physician. As a CoC-accredited facility that reports to the NCDB, coding personnel utilize the Standards for Oncology Registry Entry (STORE) guidelines put forth by the American College of Surgeons and CoC for the definition of what constitutes treatment [12]. For musculoskeletal sarcoma, the treatment initiation date is defined as the earliest of the following dates: date of the first surgical procedure, date radiation started, date systemic therapy started, or date other treatment started. Registry-reported TTI was precalculated from the date of diagnosis and the date of first treatment found within the registry. A manual review of the electronic medical record of all patients was then performed to verify the accuracy of the registry-reported diagnosis, treatment dates, and treatment type. The diagnosis date was the date of histologic confirmation (i.e., biopsy). The effective treatment date was defined by the authors as the date of the planned first oncologic excision procedure, oncologic re-excision procedure, or initiation of either radiation or chemotherapy. Patients who underwent nonexcisional biopsy procedures (incisional biopsy or staging biopsy) or unplanned incomplete excision procedures (i.e., “whoops” procedures) did not meet our definition of effective treatment. Patients who underwent planned oncologic excision procedures but had positive margins were considered to meet our definition of effective treatment. A planned positive margin procedure was interpreted based on clear documentation of the treating surgeon's forethought of margin compromise. Effective TTI was then calculated using the manually extracted dates of diagnosis and treatment. For patients with a discordant TTI value between the registry and the manual calculation (effective TTI), the reason for discordance was documented.

### 2.3. Statistical Analysis

An analysis of TTI accuracy was separately performed for patients with a registry-reported TTI = 0 days (Table 1) and for patients with a registry-reported TTI >0 days (Table 2). Registry-reported and effective TTI were compared using the Wilcoxon rank-sum test. Annual changes in TTI were calculated using a simple linear regression. The mean TTI between cohorts over the ten-year span was compared using an ANOVA test with Bonferroni pairwise comparison. All tests were two-sided, with an alpha level of 0.05. *P* values less than 0.05 were considered significant. Statistical analyses were completed with Stata software (version 16.1, College Station, Texas, USA).

### 2.4. Study Cohort Characteristics

Of the 402 patients in the cohort, the mean patient age was 55 years (range 1–95). The cohort was mostly white (87%, 349/402) and male (56%, 226/402). Nearly half of the participants were privately insured (48%, 193/402). The most common sarcoma subtype was undifferentiated pleomorphic sarcoma (30%, 121/402), and the majority were in the lower extremity (58%, 235/402). There were 118 patients (29%) who had a registry-reported TTI of 0 days (TTI = 0 cohort), while the remaining 284 patients (71%) had a registry-reported TTI >0 days (TTI >0 cohort). Most patients (70%, 280/402) were reported as being treated initially with surgery. All patients in the TTI = 0 cohort (118/118) had surgery reported as first treatment, while 57% of patients in the TTI >0 cohort (162/284) had surgery reported as first treatment. Our healthcare system is quaternary in nature with a main campus facility in an urban setting where our sarcoma team predominantly resides and ten surrounding regional hospitals. There exists a robust multidisciplinary sarcoma team consisting of orthopaedic oncology, general surgical oncology, thoracic surgery, head and neck surgery, spine surgery, radiation oncology, medical oncology, musculoskeletal radiology, and bone and soft tissue pathology. From a surgical standpoint, there exists a collegial working relationship where orthopaedic oncology treats extremity and trunk sarcoma, and surgical oncology treats intraabdominal sarcoma; retroperitoneal sarcoma is most often treated by surgical oncology, and chest wall sarcoma is most often treated by thoracic surgery. Forty-eight percent of patients (192/402) received their initial treatment from a physician involved in the sarcoma practice in our healthcare system. 33% percent of patients (132/402) received their initial treatment from a nonsarcoma physician in our healthcare system, and the remaining 19% of patients (78/402) were first treated by a physician from an outside healthcare system. Registry-reported patient, tumor, and treatment characteristics can be found in Table 3.

## 3. Results

### 3.1. TTI = 0 Cohort

In patients with a registry-reported TTI = 0 days (Table 1), 74% (87/118) of patients had a diagnostic surgical procedure (that did not complete their surgical oncologic care) coded as their first treatment. Seventy-three patients (62%) underwent an unplanned incomplete excision, and 14 patients (12%) had an incisional biopsy. After adjusting the treatment date to that of their first effective treatment received, the mean TTI increased to 43.5 ± 43.2 days (range 0–253) in the TTI = 0 cohort (*P* < 0.001) and rose to 60.4 ± 38.3 days (range 13–253) among patients who underwent an unplanned incomplete excision (*P* < 0.001). Patients who underwent an unplanned incomplete excision at an outside health system trended toward a longer effective TTI than patients first treated within our sarcoma group (61.8 days vs. 30.5 days; *P* = 0.06). Patients who underwent an unplanned incomplete excision by physicians within our health system but who were not an active part of our sarcoma team also trended toward a longer effective TTI than patients first treated within our sarcoma group (60.2 days vs. 30.5 days; *P* = 0.17). Patients who underwent an unplanned incomplete excision by physicians within our health system but who were not an active part of our sarcoma team had no different effective TTI compared to patients first treated at an outside health system (60.2 days vs. 61.8 days; *P* = 0.99).

### 3.2. TTI >0 Cohort

In patients with a registry-reported TTI >0 days (Table 2), only 4% (12/284) of patients had a discordant TTI value. Eight patients (8/284, 3%) had a biopsy procedure (including index biopsy of the primary tumor site, repeat biopsy of the primary tumor site, or biopsy of a different site, see Table 2 footnote) reported as first treatment, and four patients (4/284, 1%) were found to have a misreported date of first treatment (i.e., coder error). For those incorrectly reported, the mean registry-reported TTI was 40.3 ± 53.7 days (range 3–188), and the mean effective TTI was 33.4 ± 17.4 days (range 8–76), with a TTI difference of 6.9 days (*P*=0.27). In the entire TTI >0 cohort, the mean registry-reported TTI was 31.3 ± 26.0 days (range 2–188), and the mean effective TTI was 31.0 ± 23.9 days (range 2–141), with a TTI difference of −0.3 days (*P*=0.77).

### 3.3. TTI in the Entire Cohort and Over Time

**Figure 2:**
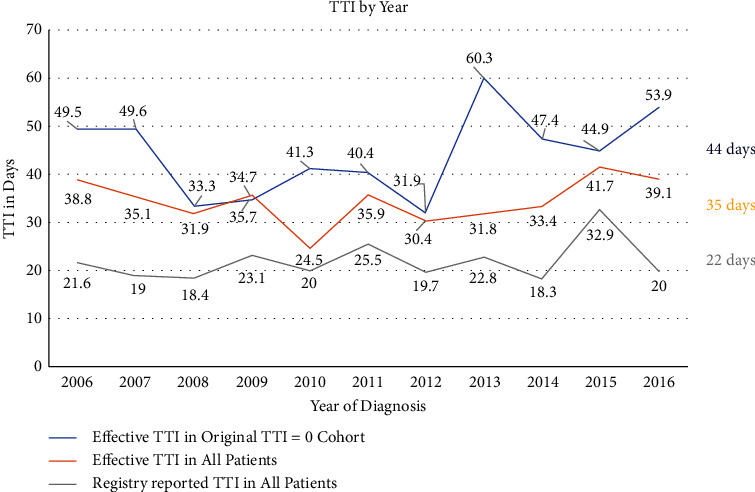
Registry-reported versus effective time to treatment initiation (TTI) by year of diagnosis. Overall difference between groups, *P* < 0.001; difference between registry-reported (gray) and effective TTI (orange) in all patients, *P* < 0.001; difference between registry-reported TTI in all patients (gray) and effective TTI in the original TTI = 0 cohort (blue), *P* < 0.001; difference between effective TTI in all patients (orange) and effective TTI in the original TTI = 0 cohort (blue), *P*=0.02. The mean TTI from 2006 to 2016 is listed next to the associated curve (44 days, 35 days, and 22 days).

Figure 2 demonstrates no significant change in TTI during the ten-year span in overall registry-reported TTI (*P*=0.29), overall effective TTI (*P*=0.46), or effective TTI in the original TTI = 0 cohort (*P*=0.47). The mean overall registry-reported TTI was 22.1 ± 26.1 days (range 0–188), while the mean overall effective TTI was 34.7 ± 31.3 days (range 0–253). In the original TTI = 0 cohort, the mean effective TTI was 43.5 ± 43.2 days (range 0–253). The differences found in the collective means between the three groups are shown in Figure 2. Patients initially cared for by our sarcoma team had the shortest effective TTI (27.6 ± 22.7 days (range 0–141)) compared to those treated within our institution though outside the sarcoma group (35.8 ± 34.3 days (range 0–190)) or at outside institutions (50.2 ± 38.1 days (range 0–253)) (*P* < 0.001).

## 4. Discussion

The purpose of this study was to assess the accuracy of TTI data in musculoskeletal soft tissue sarcoma patients reported in a large CoC-accredited cancer center's tumor registry. We compared the registry-reported TTI value (where the first treatment date was defined by STORE guidelines) to the manual calculation of the effective TTI value (where the first treatment date was defined by the authors as to when effective treatment was initiated). The registry-reported TTI differed from the effective TTI in 25% of patients (99/402). In patients with a TTI reported as greater than 0 days in the registry, there were few discordant TTI values (4%), and the reported TTI and effective TTI were nearly the same. In contrast, in patients with a TTI reported as equal to 0 days in the registry, there were discordant TTI values in nearly 75% of patients, with 84% of those being unplanned incomplete excisions and 16% being incisional biopsies, all surgical event discrepancies. These data primarily highlight the common misclassification of diagnostic procedures (unplanned incomplete excision procedures and incisional biopsies) coded as the first treatment event in this patient population. The purpose of reporting the findings of this investigation is not at all to question the diligence of those coding or abstracting the data (as coder error was impressively low at 1%), but rather to propose an alternative definition of what constitutes first surgical treatment.

These data suggest an underestimation of TTI values reported from a large cancer center registry to the NCDB. After manual review of patients' records who had undergone unplanned incomplete excision procedures (18%, 73/402), effective TTI was over 8 weeks longer on average (60 days). This discordance served as the main driver for the two-week difference found between registry-reported TTI (22 days) and effective TTI (35 days) for the overall dataset, as reported in Figure 2. These data demonstrate that the overreporting of TTI = 0 is prevalent and has a significant impact on overall TTI measurement for an institution. The second most common reason for TTI discrepancy within this dataset was an incisional biopsy being coded as a first treatment event. This, however, is inappropriate, as this is a diagnostic (not therapeutic) procedure that simply takes place in an operating room or procedural suite.

Our data showed only four of 402 patients (1%) had a misreported first treatment date by coders, leading to a discordant TTI value. It is important to note that the inappropriate coding of unplanned incomplete excision procedures or incisional biopsies is secondary to the national definition of what constitutes treatment, not coder error. The American College of Surgeons and the Commission on Cancer's STORE guidelines for musculoskeletal sarcoma state that the treatment initiation date, or “date of the first course of treatment,” is defined as the earliest of the following dates: date of the first surgical procedure, date radiation started, date systemic therapy started, or date other treatment started [12]. Given this, it is understandable how an unplanned incomplete excision or incisional biopsy would be included within this broad definition, as they are technically surgical procedures. While it may be argued that this investigation is limited to a single institution's dataset, it is reasonable to assume other institutions may see a similar pattern in their registry-reported data as these definitions are standard nationwide. Moreover, this may not only apply to soft tissue sarcoma but to many cancer types in which surgical excision is part of standard management. Of note, when radiation or chemotherapy was the registry's reported first treatment (122/402, 30%), there were no instances of TTI discrepancy. We, therefore, would argue that the definition of the “first surgical procedure” within the STORE guidelines needs to be better refined to increase the accuracy of TTI measurements. We suggest considering the term “first surgical procedure with oncologic excision: either negative margin excision or planned positive margin excision.”

From a coding standpoint, it is important to note that capturing these procedures accurately will not occur simply by using the International Classification of Diseases (ICD) or Current Procedural Terminology (CPT) codes. It may require reviewing an operative report, a pathology report, and, ideally, postoperative multidisciplinary sarcoma tumor board documentation that comments on the oncologic efficacy of the surgery. As coders are highly trained and familiar with cancer surgery terminology, the authors feel achieving accuracy with a new, more nuanced definition of the surgical procedure is only a matter of updating coder education.

Patients initially cared for by our sarcoma team had the shortest effective TTI (27 days) compared to those treated within our institution but outside our sarcoma group (36 days) or at outside institutions (50 days). This appeared to be secondary to the fact that 67% (49/73) of unplanned incomplete excision procedures were performed by outside institutions and that 30% (22/73) were performed by nonsarcoma physicians in our institution (compared to only 3% (2/73) of unplanned incomplete excision procedures performed by surgeons within our sarcoma group). This lends further evidence in support of early referral to a multidisciplinary sarcoma team to avoid a “whoops” procedure that has been shown to have higher rates of amputation, the necessity for plastic surgery reconstruction, and the cost incurred [9, 13]. In an effort to increase education to prevent “whoops” procedures, prior work in the United Kingdom coined the well-known mantra that “if your lump is bigger than a golf ball and growing, think sarcoma” [14, 15]. Other evidence-based practices to minimize “whoops” procedures are obtaining preoperative advanced imaging and a biopsy for suspicious masses. In 2018, Mesko et al. reported that nearly 40% of 397 soft tissue sarcoma patients over a nine-year span underwent incomplete excision surgery [8]. In those, 42% had preoperative advanced imaging and only 16% had a preoperative biopsy, compared to 91% and 85% in the wide excision group, respectively. As the present data demonstrate that 18% of patients (73/402) underwent “whoops” procedures, it is evident that continued national education is necessary. Interestingly, in soft tissue sarcoma, it has also been shown that local recurrence, metastasis-free survival, and overall survival are not negatively impacted if adequate re-excision and multidisciplinary treatment are performed after an unplanned incomplete excision [9]. This lack of negative prognostic effect following adequate re-excision surgery supports the notion that has been previously shown that quality of care, rather than timeliness of care, may have a larger impact on prognosis in soft tissue sarcoma [4, 16]. If TTI is felt to be an important quality metric in sarcoma care, it is necessary that it be reported with the clearest understanding of its meaning so that any potential association with prognosis can be identified and properly interpreted. This study does not aim to disqualify the rationale for reporting TTI but rather amplify it through careful reconsideration of its definition surrounding surgical events to further improve the accuracy of the data used to determine TTI's overall significance as a quality metric.

This is not the first reported instance in the sarcoma literature of discordance between databases or registries and manual review of patient data. Holt et al. in 1998 reported only 60% agreement in seven fundamental demographic variables (such as birthdate, medical record number, and presence or absence in the registry) between two institutional administrative databases (billing and clinical) and a physician-kept log [17]. More recently, Lyu et al. showed only 61% of over 1,200 sarcoma patients treated at a large cancer center were accurately coded as having a sarcoma diagnosis when compared to pathology reports [18]. They conclude that the heterogeneity of histologic sarcoma subtypes (unique soft tissue sarcoma histologies now exceed 50) and variable nomenclature likely play a role in discordance and that their findings are not likely isolated to their institutions alone, as tumor registrars across the country are trained by the American Joint Committee on Cancer guidelines [18, 19].

There are limitations to this study. First, this analysis was performed at a single tertiary cancer center. Though the assumption that similar findings may be seen at other institutions is reasonable, it is not guaranteed. Second, outside of the TTI-related variables (TTI, date of diagnosis and treatment, type of treatment, and provider type), we did not manually evaluate the coding accuracy of other demographic variables in this analysis. As well, due to the limitations of the dataset, we were unable to evaluate if oncologic outcomes (local recurrence, distant spread, and mortality) were associated with discordance between registry-reported TTI and effective TTI or the occurrence of a “whoops” procedure. Undoubtedly, this additional analysis would help further characterize the clinical implications of TTI, though other assessments of TTI in large databases have been performed prior [3, 4].

## 5. Conclusion

This internal review of a single institution's sarcoma registry suggests an underestimation of TTI secondary to an all-inclusive definition of what constitutes surgical treatment and may shed light on the nature of TTI data collected by the NCDB. Nearly 75% of patients with a reported TTI of 0 days had a diagnostic surgical procedure coded as their first treatment event. Unplanned incomplete excision procedures and incisional biopsies were the most common reasons for TTI discordance, and upon manual review of medical records, the effective TTI for these patients was greater than 8 weeks longer than reported. This misclassification of unplanned incomplete excision procedures and incisional biopsies as surgical treatment procedures is secondary to the current national definition of what constitutes surgical treatment, not coder error. A working group between the CoC, ACS, and partner institutions aimed at clarifying the definition of what constitutes surgical treatment may be warranted to assure that the most accurate data are collected and reported when assessing TTI in musculoskeletal soft tissue sarcoma.

## Figures and Tables

**Table 1:** Treatment characteristics of patients with a registry-reported TTI = 0 days.

	*N* (%)	Effective TTI, days (SD) [range]^*∗*^	*P* value
Total TTI = 0 cohort	118 (100)	43.5 (43.2) [0–253]	<0.001
Correctly reported TTI = 0	31 (26)	0	—
First treatment performed by			—
Sarcoma physician *ϕ*	9 (8)	0	—
Nonsarcoma physician	20 (17)	0	—
Outside health system	2 (2)	0	—
Treatment type			
Excisional biopsy	31 (26)	0	—
Incorrectly reported TTI = 0	87 (74)	59.1 (40.1) [5–253]	<0.001
Unplanned incomplete excision reported as first treatment	73 (62)	60.4 (38.3) [13–253]	<0.001
First treatment performed by			
Sarcoma physician *ϕ*	2 (2)	30.5 (3.5) [28–33]	0.33
Nonsarcoma physician	22 (19)	60.2 (34.2) [13–138]	<0.001
Outside health system	49 (42)	61.8 (40.6) [14–253]	<0.001
First effective treatment			
Re-excision	59 (50)	59.5 (40.0) [13–253]	<0.001
Radiation	13 (11)	66.8 (30.6) [27–138]	<0.001
Chemotherapy	1 (1)	31	—
Incisional biopsy reported as first treatment	14 (12)	51.9 (49.5) [5–190]	<0.001
First treatment performed by			
Sarcoma physician *ϕ*	6 (5)	29.5 (30.2) [5–87]	0.002
Nonsarcoma physician	4 (3)	88 (76.3) [27–190]	0.01
Outside health system	4 (3)	49.2 (23.2) [28–74]	0.01
First effective treatment			
Excision	9 (8)	46.9 (35.6) [5–103]	<0.001
Radiation	3 (3)	28.7 (11.4) [16–38]	0.04
Chemotherapy	2 (2)	109 (114) [28–190]	0.10

^*∗*^Data reported in days as mean (SD) [range]. All patients in the TTI = 0 cohort underwent surgery as registry-reported first treatment. *P* values represent a comparison between effective TTI and reported TTI of 0 days in all patients. *ϕ* = sarcoma physician and nonsarcoma physician providers were within our health system.

**Table 2:** Treatment characteristics of patients with a registry-reported TTI >0 days.

	*N* (%)	Reported mean TTI^*∗*^	Effective mean TTI^*∗*^	TTI difference^#^	*P* value
Total TTI >0 cohort	284 (100)	31.3 (26.0) [2–188]	31.0 (23.9) [2–141]	−0.3	0.77
Correctly reported TTI >0 cohort	272 (96)	30.9 (24.2) [2–141]	—	—	—
First treatment performed by					
Sarcoma physician *ϕ*	165 (58)	28.6 (22.6) [3–141]	—	—	—
Nonsarcoma physician	84 (30)	35.5 (27.4) [2–140]	—	—	—
Outside health system	23 (8)	30.2 (21.5) [2–108]	—	—	—
First effective treatment					
Excision	150 (53)	30.8 (25.5) [2–141]	—	—	—
Radiation	86 (30)	32.5 (21.6) [3–140]	—	—	—
Chemotherapy	36 (13)	26.9 (24.7) [2–108]	—	—	—
Incorrectly reported TTI >0 cohort	12 (4)	40.3 (53.7) [3–188]	33.4 (17.4) [8–76]	−6.9	0.27
Biopsy procedure reported as first treatment^^^	8 (3)	27.8 (31.6) [3–85]	29.0 (9.9) [8–41]	1.2	0.20
Date of first treatment misreported	4 (1)	65.5 (83.5) [12–188]	42.3 (26.9) [20–76]	−23.2	0.69

^*∗*^Data reported in days as mean (SD) [range]. *P* values represent a comparison between effective TTI and reported TTI. *ϕ* = sarcoma physician and nonsarcoma physician providers were within our health system. # = TTI difference in days between reported TTI and effective TTI. ^ = index biopsy of the primary tumor site reported as first treatment (2 patients); repeat biopsy of the primary tumor site after nondiagnostic index biopsy reported as first treatment (5 patients); biopsy of a different site reported as first treatment (1 patient). In 7 patients with an index or repeat biopsy reported as first treatment, a new diagnosis date was calculated to determine effective TTI.

**Table 3:** Registry-reported patient, tumor, and treatment characteristics.

	*N* (%)
Total cohort	402 (100)
Age (yrs)	
<18	17 (4)
18–40	84 (21)
41–70	199 (49)
71+	102 (26)
Age, mean yrs (SD)	55 (20.1)
Sex	
Male	226 (56)
Female	176 (44)
Race	
White	349 (87)
Black	44 (11)
Other	9 (2)
Insurance status	
Private	193 (48)
Medicare	116 (29)
Medicaid	23 (6)
Uninsured	14 (3)
Military	2 (1)
Unknown	54 (13)
State of residence	
Ohio	157 (39)
Other state	245 (61)
Histology	
Undifferentiated pleomorphic sarcoma	121 (30)
Liposarcoma	63 (16)
Myxofibrosarcoma	51 (13)
Synovial sarcoma	28 (7)
Leiomyosarcoma	26 (6)
Other	24 (6)
Malignant peripheral nerve sheath tumor	22 (5)
Extraskeletal bone sarcoma	17 (4)
Rhabdomyosarcoma	15 (4)
Dermatofibrosarcoma protuberans	12 (3)
Angiosarcoma	9 (2)
Round cell sarcoma	7 (2)
Clear cell sarcoma	7 (2)
Location	
Lower extremity	235 (58)
Upper extremity	69 (17)
Pelvis	38 (9)
Chest wall	34 (9)
Trunk	26 (7)
Grade	
Grade 1	41 (10)
Grade 2	65 (16)
Grade 3	130 (32)
Grade 4	57 (14)
Unknown	109 (28)
Stage	
I	95 (24)
II	90 (22)
III	116 (29)
IV	39 (10)
Unknown	62 (15)
Time to treatment initiation (TTI) cohorts	
TTI = 0 cohort	118 (29)
TTI >0 cohort	284 (71)
First treatment type	
Surgery	280 (70)
Radiation	86 (21)
Chemotherapy	36 (9)
First treatment type by TTI cohort	
TTI = 0	118
Surgery	118 (100)
TTI >0	284
Surgery	162 (57)
Radiation	86 (30)
Chemotherapy	36 (13)
Physician type who performed first treatment	
Sarcoma physician within our health system	192 (48)
Nonsarcoma physician within our health system	132 (33)
Outside health system	78 (19)

SD = standard deviation.

## Data Availability

The data used to support the findings of this study are available from the corresponding author upon request.
